# Predicting wear behavior of AZ31/TiC composites produced via ultrasonic vibration assisted friction stir processing using machine learning models

**DOI:** 10.1038/s41598-026-44372-0

**Published:** 2026-03-24

**Authors:** T. Satish Kumar, S. Shalini, Jana Petrů, M. K. Mishra, Kanak Kalita

**Affiliations:** 1 Department of Mechanical Engineering, Amrita School of Engineering, Amrita Vishwa Vidyapeetham, Coimbatore, 641112 India; 2 Department of Physics, PSG Polytechnic College, Coimbatore, India; 3 Department of Machining, Assembly and Engineering Metrology, Faculty of Mechanical Engineering, VSB-Technical University of Ostrava, 70800 Ostrava, Czech Republic; 4 Department of Metallurgical and Materials Engineering, Malaviya National Institute of Technology Jaipur, Jaipur, 302017 India; 5 Department of Mechanical Engineering, Rajalakshmi Institute of Technology, Chennai, 600124 India

**Keywords:** AZ31 alloy, TiC, FSP, Ultrasonic vibration, Hardness, Machine learning, Wear prediction, Engineering, Materials science

## Abstract

This work aims to produce AZ31 Mg alloy reinforced with 15 vol.% TiC utilizing a unique Friction Stir mechanical vibration Processing (FSVP) technology, emphasizing the improvement in tribological properties of the produced composite. The dry sliding wear characteristics of the composite generated using conventional Friction Stir Processing (FSP) and the suggested FSVP process were assessed under different applied loads utilizing a pin-on-disc tribometer. The experimental findings indicate that FSVP markedly decreases the coefficient of friction and enhances wear resistance, especially under elevated load circumstances. The incorporation of 15 vol.% TiC into the AZ31 matrix reduced the wear rate by approximately 25% at moderate loads and up to 50% at elevated loads. Analysis of the wear mechanisms indicated a progression from mild oxidative and abrasive wear at lower loads to delamination, adhesion and severe plastic deformation under higher load regimes. The superior wear performance of the FSVP-processed AZ31/15 vol.% TiC composites is attributed to increased surface hardness and enhanced work-hardening behavior. Furthermore, a Gradient Boosting machine learning model exhibited excellent predictive accuracy achieving R^2^ values of 0.9925.

## Introduction

Metal matrix composites (MMCs), particularly magnesium (Mg)-based systems, have gained prominence in tribological and lightweight structural applications due to their low density, high specific strength and excellent wear resistance^1–3^. Owing to these advantages, Mg alloys are increasingly replacing aluminum in automotive subsystems such as transmission casings, engine blocks and chassis components^4,5^. The wear performance of Mg alloys can be tailored through the addition of hard ceramic reinforcements like TiC, SiC, TiB₂, Al₂O₃ and HEA which enhance their load-bearing capacity and reduce material degradation under sliding contact^6–9^.

Among these, titanium carbide (TiC) is particularly promising due to its high hardness, chemical inertness and thermal stability. Several studies have reported the enhancement of hardness, tensile strength and wear resistance in TiC-reinforced Mg and AZ-series alloys^10–13^. For instance, Kaliyaperumal et al.^14^ demonstrated that AZ31 alloys reinforced with 6–8 wt.% TiC exhibited up to 14.6% improvement in tensile strength and a 40% reduction in wear rate, although higher TiC content led to brittleness and particle agglomeration. Similarly, Aydin et al.^15^ and Kumar et al.^16^ found that AZ91-based TiC composites exhibited reduced wear and coefficient of friction (COF) but showed non-uniform distribution and interfacial decohesion at higher reinforcement levels.

To address these dispersion challenges, friction stir vibration processing (FSVP) has emerged as an effective technique. Unlike conventional friction stir processing (FSP), FSVP incorporates ultrasonic vibrations during material stirring, leading to refined grains, enhanced particle distribution and reduced porosity through mechanisms such as acoustic softening, cavitation-induced mixing and dynamic recrystallization^7,8^. Zhou et al.^9^ concluded that the application of vibration during FSP significantly improved the dispersion of FeAlCrMoNb HEA particles in AZ31 alloy, leading to refined grain structure. As a result, enhanced hardness, strength and wear resistance were achieved compared to conventional FSP. Bagheri et al.^10^ observed enhanced hardness and more homogeneous reinforcement dispersion when nano-SiC were introduced into AZ91 via FSVP. These improvements are attributed to the combined effects of thermal–mechanical and acoustic energy, which promote microstructural refinement and mitigate clustering of reinforcements.

Despite these developments, predictive understanding of tribological behavior under varying operational conditions (load, speed, reinforcement %) remains empirical. To overcome this, machine learning (ML) has been increasingly applied in materials science to model complex, nonlinear relationships among process parameters and material properties. ML can provide accurate, generalizable models to predict wear, hardness and COF without exhaustive physical testing.

Recent research has demonstrated the potential of machine learning (ML) techniques in predicting and optimizing the tribological behavior of composite materials. For instance, Mukunda et al.^17^ applied XGBoost regression to predict the wear characteristics of AZ31/MWCNT composites under varying sliding speeds and loads, achieving a coefficient of determination (R^2^) of 0.999 for training data and 0.908 for testing, indicating strong model performance. Ammisetti and Kruthiventi^18^ investigated the wear behaviour of AZ91 magnesium composites reinforced with Al₂O₃ and graphene fabricated via stir casting. Qualitative analysis showed that applied load and sliding conditions-controlled wear, while reinforcement addition enhanced wear resistance through improved load-bearing capacity. Among the evaluated ML models (DT, RF and GBR), the GBR model exhibited superior predictive capability, achieving high accuracy with R^2^ = 98.89 and RMSE = 0.1996. Similarly, Sheikh et al.^19^ investigated Al5052-cenosphere composites and observed that increasing the reinforcement content enhanced both microhardness and wear resistance. Among the four ML models evaluated, Random Forest demonstrated the best balance between prediction accuracy (R^2^ = 0.94) and generalizability. Feature importance analysis identified reinforcement weight percentage as the most influential parameter affecting wear behavior. Collectively, these studies highlight the efficacy of ML-based methodologies in accelerating the design and development of advanced composites by minimizing experimental costs and uncovering complex, multidimensional relationships within the data.

The present study investigates the wear and microhardness behavior of AZ31/TiC composites fabricated through both conventional Friction Stir Processing (FSP) and advanced FSVP. Composites were developed using 15% TiC reinforcement. It is hypothesized that ultrasonic vibration in FSVP will improve reinforcement dispersion and grain refinement, leading to enhance wear resistance. To complement the experimental findings, we employ supervised ML models—including Random Forest, Linear Regression and Gradient Boosting—to predict the wear rate and coefficient of friction based on input features such as load, sliding velocity and TiC content. The models’ predictive accuracy is evaluated using the coefficient of determination (R^2^) and error metrics such as RMSE and MAE. Feature importance analyses are also performed to identify the most influential parameters governing wear performance. This dual experimental–computational approach provides both physical insights and a predictive framework for the design of wear-resistant Mg-based composites.

## Materials and manufacturing process

The present investigation used AZ31 Mg alloy plates of 100 × 50 × 8 mm as the matrix material, reinforced with 15 vol.% TiC particles, with a particle size ranging from 4 to 6 µm. Before the integration of TiC, a sequence of preliminary processes was carefully conducted. Grooves were machined into the AZ31 plates to enable the integration of the reinforcing particles in the matrix. The TiC powders were systematically inserted into the grooves, which were then sealed with a pin-less technique to effectively encapsulate the particles. The specimens were subsequently processed using both standard FSP and FSVP. The process employed an H13 tool steel stirrer, featuring a tilt angle of 2° and an axial load of 4 kN. A uniform traverse speed of 100 mm/min and a rotational speed of 900 rpm were employed for both FSP and FSVP. Ultrasonic vibrations during FSVP were produced using a magneto strictive transducer (RELTEC, Russia) equipped with a stainless steel (SS304) horn. The ultrasonic waveguide was incorporated into the FSP fixture as outlined in a prior report^20^. Samples were subjected to ultrasonic vibration during FSP using 1.5 kW of power input and a constant frequency of 20.1 kHz, with vibration amplitude of 0.01 mm were consistently utilized during the FSVP^21^. The vibration was transmitted through rigid clamping at the backing plate, ensuring effective coupling between the vibration source and the workpiece. The incorporation of ultrasonic vibrations in FSP enhances material flow and promotes intense dynamic recrystallization through acoustic softening and cavitation effects. This results in significant grain refinement, improved homogeneity of intermetallic phase distribution and enhanced mechanical and corrosion properties of the processed alloy^22^.

The density of the produced AZ31/TiC composites was determined experimentally by Archimedes’ principle. The samples were measured on a high-precision digital balance with an accuracy of 0.001 g. Initially, the samples were weighed in air (*W*_*a*_) and subsequent measurements were made by submerging the samples in distilled water (*W*_*w*_). Equation (1) was then used to calculate experimental density.

1 $$\rho_{{{\mathrm{exp}}}} = \frac{{w_{a} }}{{w_{a} - w_{w} }} \times \rho_{w}$$

where ${\rho }_{\mathrm{exp}}$ is the measured density and ${\rho }_{w}$ is the density of water. Theoretical density was calculated based on the law of mixtures using Eq. (2).

2 $$\frac{1}{{\rho_{th} }} = \frac{{1 - w_{r} }}{{\rho_{m} }} + \frac{{w_{r} }}{{\rho_{r} }}$$

Theoretical density, (*ρ*_*th*_) was calculated using the mass fraction of reinforcements (*w*_*r*_) and the densities of the matrix (*ρ*_*m*_) and reinforcements (*ρ*_*r*_). The porosity of the composite specimens was calculated using Eq. (3).

3 $$P = \left( {1 - \frac{{\rho_{\exp } }}{{\rho_{th} }}} \right)$$

where ${\rho }_{\mathrm{exp}}$ and ${\rho }_{th}$ are the measured and calculated densities, respectively. Four specimens of each composite material were systematically assessed and the resultant average porosity was documented. Optical Microscopy (OM, Model: Leitz) and SEM integrated with EDS (Model: JEOL) were utilized to investigate the microstructural characteristics of the AZ31/TiCp composites processed through FSP and FSVP. To microstructural examination, samples were extracted from the stir zone of both FSP and FSVP specimens. Prior to the etching process, metallographic specimens underwent mechanical grinding and polishing. The etching procedure was executed using an acetic picric solution formulated with 10 ml of distilled water, 5 ml of acetic acid, 100 ml of ethanol and 6 g of picric acid. The phase composition of the FSP and FSVP samples was examined using X-ray diffraction (XRD) with a Philips X’Pert Pro diffractometer, employing Cu-Kα radiation (λ = 1.54056 Å) at a rate of scanning of 2°/min.

The microhardness of the synthesized composites was assessed using a Vickers microhardness tester (Mitutoyo) with a consistent load of 100 g applied for 10 s. To evaluate the tribological performance of the AZ31 alloy and its composites, dry sliding wear tests were performed following ASTM G99-95a standards using a Ducom (Bangalore) pin-on-disc apparatus with a disc track radius of 10 mm. All experimental trials were performed at ambient temperature (33 ± 2 °C) under arid conditions. The wear tests were conducted at sliding velocities of 1, 2 and 3 m/s over a fixed sliding distance of 1500 m under applied loads of 10, 20, 40, 60 and 80 N. EN31 steel with a hardness of 62 HRC and a polished surface finish (Ra ≈ 0.01 µm) was used as the counterface material. Prior to testing, the composite samples were polished to achieve an average surface roughness of Ra ≈ 0.05 µm to ensure consistent contact conditions during the wear experiments. Each test was conducted in triplicate and the average value was presented as the conclusive wear rate.

**Fig. 1:**
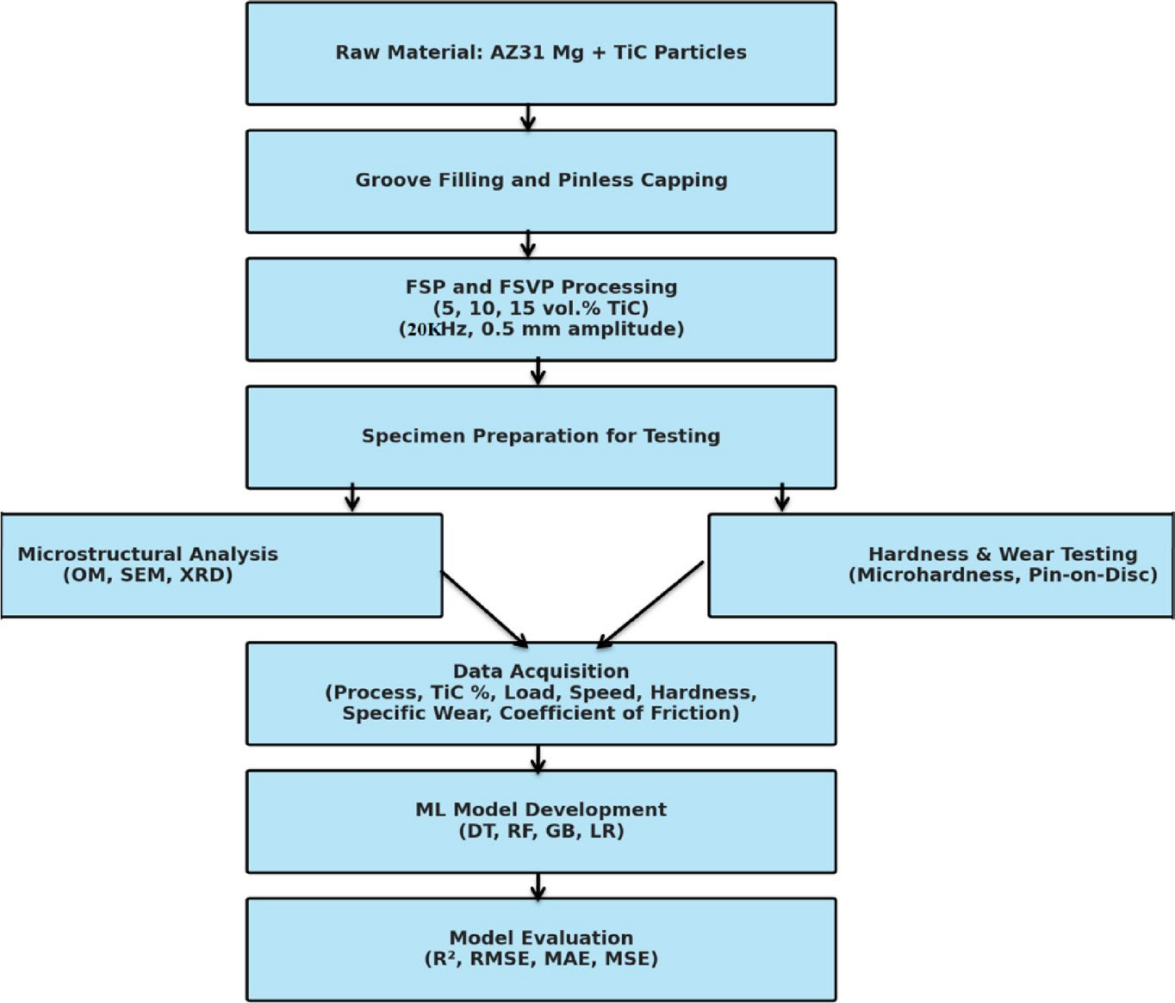
Overview of the experimental pipeline and machine learning workflow.

Figure 1 illustrates the experimental methodology and machine learning (ML) workflow adopted in this study. Integrated sensors continuously monitored the coefficient of friction throughout the duration of the test. Wear mass loss was computed by assessing the weight differential of the samples pre- and post-testing utilizing a computerized digital balance with an exactness of 0.1 mg. The final wear rate was ascertained through averaging the outcomes from three specimens. The measured mass loss was then used to calculate the volume loss. The volumetric wear rate was calculated by dividing the measured volume loss by the sliding distance. The specific volumetric wear rate was further obtained by normalizing the volume loss with respect to the applied load and sliding distance. Sliding speed was calculated as V = 2πrN/60, where *r* is disc radius and *N* is rotational speed (rpm). SEM was employed to scrutinize the worn surfaces to facilitate a detailed analysis of the wear mechanisms.

## Results and discussion

### Microstructural evaluation

**Fig. 2:**
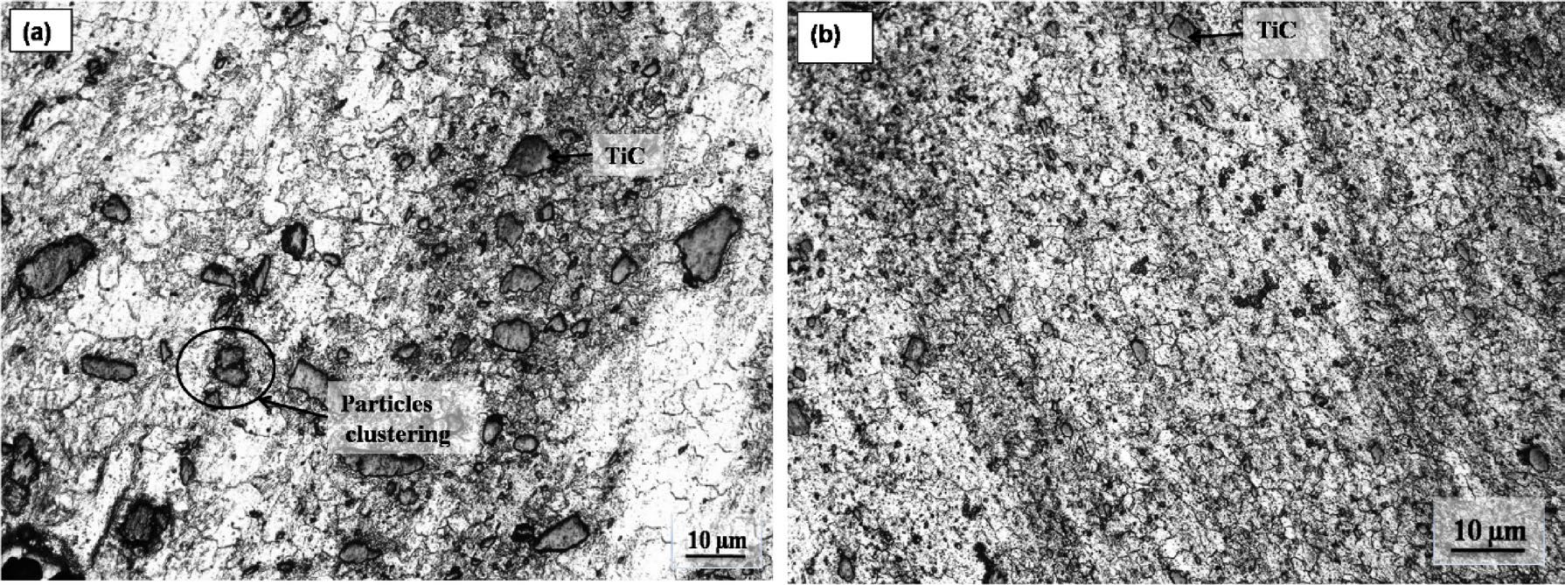
Microstructure of AZ31/15 vol.% TiC composites produced via (**a**) FSP and (**b**) FSVP.

**Fig. 3:**
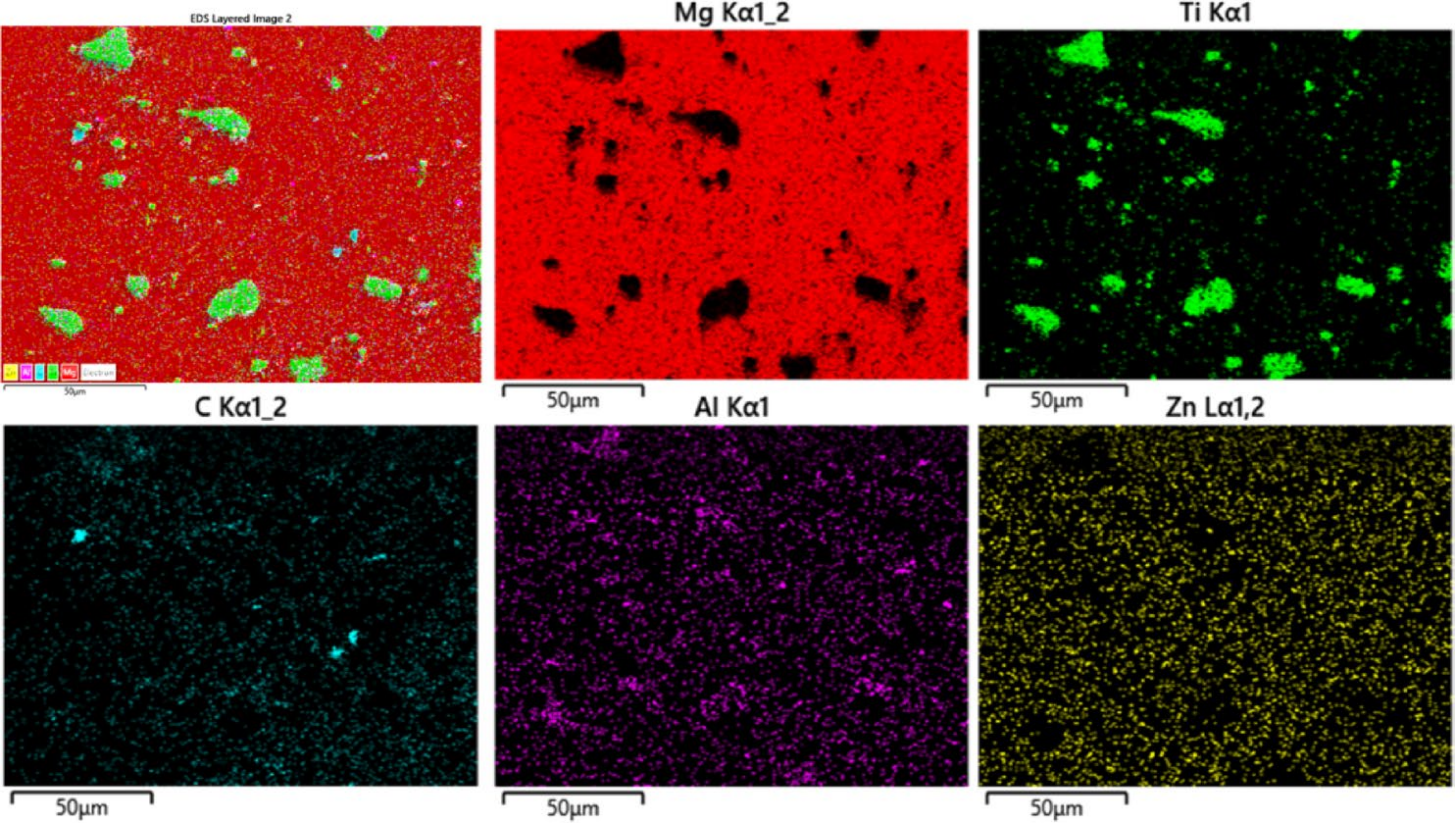
Elemental distribution within AZ31/15 vol% TiC FSVP surface composites.

Figure 2 shows the microstructure of AZ31/15 vol.% TiC composites produced via FSP and FSVP. Despite the micrographs’ precise spot location, it is notable that the TiC particles are steadily distributed in the composites. A few areas of the composites produced by traditional FSP (Fig. 2a) show the clustering of TiC particles. In contrast as shown in Fig. 2b, samples produced by FSVP showed consistent distribution of reinforcement within SZ. The TiC particles are found to be surrounded by the AZ31 alloy matrix and no other compounds are observed to form at the interface. The microstructure of FSVP AZ31/15 vol.% TiC composite has no discernible pores and the TiC particles are uniformly distributed. The absence of undesirable substances such as Mg oxide or Mg diboride suggests that the fabrication process for AZ31-TiC by FSP composites was successful. It is evident that the TiC particles are suitably wet by the Mg matrix without passing through any chemical reaction and after FSP and FSVP, these particles formed a strong bonding with the AZ31 matrix. FSVP is attributed to the fragmentation of coarse particles during the friction stir process. Additional information about the mechanism of grain refinement has been previously reported^9,23^. However, compared to the AZ31 alloy, the FSP and FSVP AZ31-TiC composites showed lower grain sizes, suggesting that the TiC particles aided in the process of dynamic recrystallization. The AZ31 alloy is composed of large grains with an average grain size of about 60 µm. The grain size in the AZ31/15 vol% TiC FSVP MMC is further reduced to 8–10 µm with FSVP, is explained in the previous report^20^. Since TiC particles have a high melting temperature and hardness, they act as diverse nucleation sites in Mg grains restricting the grain growth. Figure 3 presents the elemental distribution of AZ31/15 vol.% TiC FSVP composites. The results indicate a uniform dispersion of TiC particles throughout the FSVP composite. The elemental mapping spectra confirm the presence of Ti, C, Mg and Al with no significant depletion observed in the TiC spectrum at the particle–matrix interface.

### Physical and mechanical properties

The porosity and density of the AZ31 alloy and TiC reinforced composites produced via FSP and FSVP samples are detailed in Table 1. The density of the AZ31 alloy augmented with the increasing concentration of TiC particles. This can be explained by the fact that TiC has a greater density (4.93 g/cm^3^) than AZ31 alloy (1.77 g/cm^3^). AZ31/15 vol.% TiC composites produced via FSVP led to a marginal improvement in the density. This can be ascribed to the excellent bonding among the TiC and the AZ31 alloy. Furthermore, porosity is noted at few places in composite samples produced via FSP. Namdev et al.^24^ have reported on comparable observations concerning AA7075-TiC-Gr surface composites synthesized via FSP. In contrast to FSP, the porosity levels in the composite samples produced through the FSVP process in this study are comparatively low. In fact, FSVP reduces porosity, increases particle/matrix wetting, reduces agglomeration and improves the spreading of particles uniformly in the matrix.

**Table 1:** Porosity and density of AZ31 alloy its composites.

Material	*ρ*_*t*_ (g/cm^3^)	*ρ*_*e*_ (g/cm^3^)	Porosity (%)
AZ31	1.772	1.730 ± 0.017	2.3
AZ31/15 vol.% TiC FSP	2.243	2.199 ± 0.020	1.96
AZ31/15 vol.% TiC FSVP	2.243	2.223 ± 0.022	0.89

**Fig. 4:**
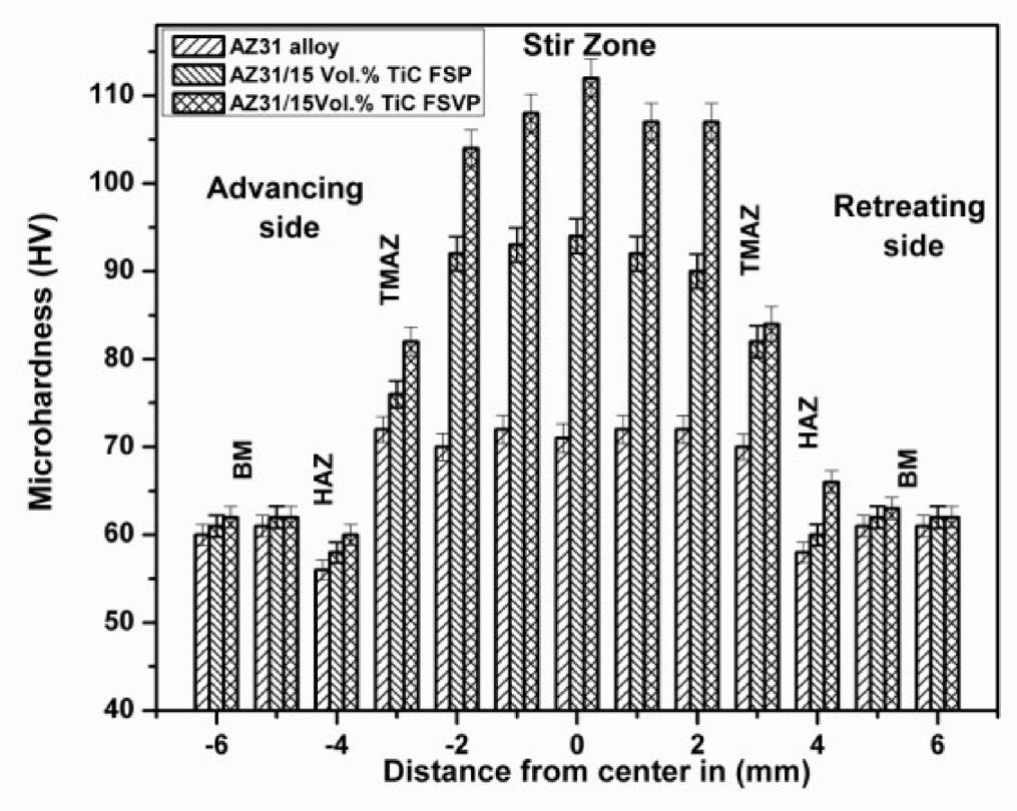
Microhardness of the AZ31 alloy and AZ31/15 vol.% TiC composites produced by FSP and FSVP.

Figure 4 depicts the hardness characteristics of AZ31 alloy, FSP and FSVP AZ31/15 vol.% TiC composites reinforcing 15 vol.% TiC particles is observed to increases the matrix hardness, from 62 HV to a maximum value of 114 HV which is an 83 % increase. The improved mechanical responsiveness of the composite samples can be attributed to several strengthening methods. The Hall-Petch effect has taken center stage as the primary strengthening mechanism because of grain refinement^25,26^. This is due to the addition of TiC particles and further by dynamic recrystallization of grains that are generated during the FSVP process. Other factors such as the load transfer effect, increased hardness of the distributed TiC particles and high dislocation density along the matrix/reinforcement has also contributed to the grain refinement.

### Friction coefficient and wear rate

**Fig. 5:**
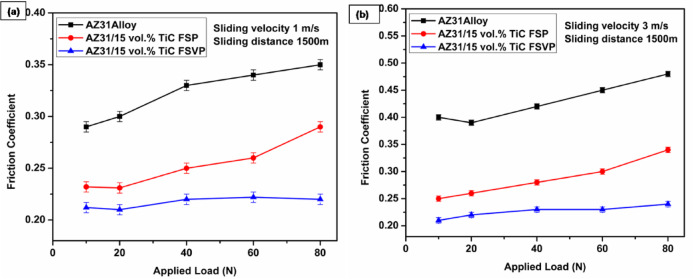
FC values for the AZ31 alloy and AZ31/TiC composites (**a**) FC vs. load at 1 m/s sliding velocity and (**b**) FC vs. load at 3 m/s sliding velocity.

The friction coefficient (FC) as a function of load for the AZ31 matrix alloy, FSP and FSVP processed AZ31/15 vol.% TiC composites under varying loads and sliding speeds is shown in Fig. 5a, b. Figure 5a displays the average FC for each tested sample under various loads through the steady wear stage. This graph reveals how the FC changes as the applied load increases for AZ31 alloy and its TiC-reinforced composites. The test uses a sliding velocity of 1 m/s and a 1500 m sliding distance. The AZ31 alloy has the highest FC for all load values, which means it faces more resistance when sliding. The AZ31/15 vol.% TiC FSP composite has a lower FC than the base alloy. The AZ31/15 vol.% TiC FSVP composite shows the lowest FC. These findings suggest that addition of TiC and usage of the FSVP processing method helped to reduce the friction. This might be because of improvement in surface properties due to the presence of TiC particles. Figure 5b shows the influence of sliding speed (3 m/s) on the FC with a sliding distance of 1500 m. When compared to the trend at 1 m/s, we clearly see the FC goes up for all materials. The AZ31 alloy still has the highest FC but the TiC-reinforced composites keep their friction lower. This upward trend tells us that faster sliding speeds lead to more intense wear, which makes the friction forces stronger. The AZ31/15 vol.% TiC FSVP composite stands out by keeping its FC lower, proving it has better wear-resistant qualities. The FC of AZ31 alloy increases with an increase in applied load under all circumstances. The contact surface between worn discs and AZ31 alloy pins is frequently in an elastoplastic state during sliding wear. It is believed that when applied stress increases, the FC rises along with the true contact area and surface roughness.

Compared to the AZ31 alloy, FSP and FSVP processed AZ31/15 vol.% TiC composites exhibited low FC even at higher loads. The development of an oxide layer or certain material changes may also have an impact on the contact conditions. As seen in Fig. 5a, b, the AZ31 alloy FC is consistently greater than AZ31/TiC composites across all wear regimes. Additionally, when subjected to applied wear stress, FSVP processed composites with higher hardness show a more stable FC. The wear property of FSVP processed composites with evenly distributed TiC particles reduces the metallic contact and adhesion between the composite pin surface and disc. The low FC and less wear rate of AZ91-B_4_C composites are supported by the findings of Singh et al.^27^ on Mg–B_4_C and Sankar et al.^28^ on AZ91–B_4_C composites, respectively.

Figure 5 shows the FC values for AZ31 alloy and AZ31/TiC composites (a) FC vs applied load at a sliding velocity of 1 m/s (b) FC vs. applied load at 3 m/s sliding velocity. At a reduced sliding velocity of 1 m/s, the FC escalates with the applied weight across all materials, adhering to a uniform pattern. AZ31 alloy has the highest FC, whilst TiC-reinforced composites display reduced friction, with the FSVP-processed composite highlighting optimal performance. The decrease in FC in TiC-reinforced samples signifies higher lubricating effects, superior wear resistance and improved dispersion of reinforcement particles. The reduced velocity yields more steady frictional behavior relative to the 3 m/s condition, accompanied by marginally lower total values. The negligible variances among the trials validate the dependability of the results, highlighting the enhanced tribological efficiency of FSVP processing.

The FC demonstrates a rising trend with the applied load for all examined materials. The AZ31 alloy exhibits the highest FC, signifying increased resistance to motion and enhanced interfacial adhesion. Conversely, the AZ31/15 vol.% TiC composite fabricated using friction stir vibration processing (FSVP) exhibits the lowest friction coefficients, underscoring its exceptional tribological performance. The AZ31/15 vol.% TiC composite, subjected to FSP exhibits moderate friction values, underscoring the reinforcement’s contribution to diminishing interfacial shear. The observed trend indicates that the inclusion of TiC markedly diminishes friction, owing to increased load-bearing capacity and enhanced surface stability, which alleviates material contact at high sliding velocities.

**Fig. 6:**
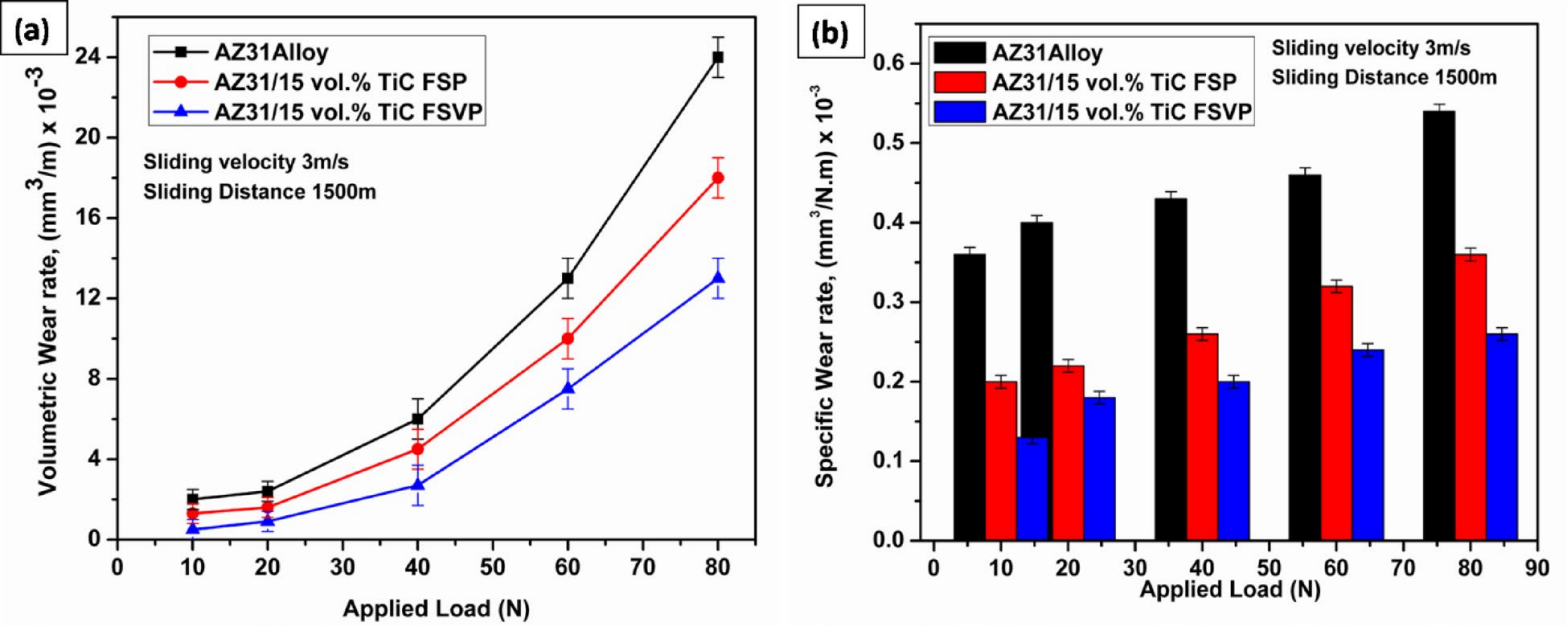
(**a**) Volumetric wear rate, (**b**) specific wear rate with respect to applied load.

Figure 6a, b depicts the volumetric wear rate and specific wear rate for the AZ31 alloy, as well as for AZ31/15 vol.% TiC composites processed using FSP and FSVP. Measurements of wear rates were made under a range of applied load, from 10 to 80 N, sliding velocity 3 m/s for a constant sliding distance 1500 m. It is seen that both the matrix and its composites wear decrease and the depth of the worn tracks increases steadily as the applied load increases. Moreover, the composites exhibited a reduced wear rate relative to the AZ31 alloy under all investigated wear regimes. In addition, the wear rate of FSVP processed AZ31/15 vol.% TiC composites decreased even more. Investigations in the past have also revealed a similar trend in variations of wear rate^8^. Notably, the AZ31/15 vol.% TiC FSVP composite performs the best, due to the homogeneous distribution of TiC particles, which help reduce severe wear. These findings suggest that TiC reinforcement, especially using the FSVP method, develops the wear resistance of AZ31 alloy by minimizing material loss under high-stress conditions.

Adding TiC makes the material last longer, with AZ31/15 vol.% TiC FSVP composite wearing out the slowest. The TiC-reinforced composite exhibits lower wear due to its enhanced load-bearing capacity and superior wear resistance, as shown in Fig. 5b. This improvement is attributed to the uniform distribution of TiC particles within the matrix, which increases the overall hardness and mitigates material loss during sliding. Consequently, the increase in wear resistance of FSVP composites can be associated with the following factors: (i) The FSVP composites has fine grains, less porosity and hence can become harder, (ii) The contact area between the corresponding pin and the composites matrix is decreased by the fine TiC particles present. (iii) The inclusion of TiC particles prevents thermally induced deformation since the matrix gets stronger as the load-bearing capacity increases. All samples showed decent wear resistance at lower average loads of 10 N and 20 N. In contrast to their matrix alloy, the composites showed superior wear resistance when exposed to greater loads of 60 N and 80 N. In comparison to the AZ31 alloy, the FSVP treated AZ31/15 vol.% TiC sample showed better wear resistance. It is found that under a higher load of 80 N, the wear rate reduced by as much as 50%. The superior strength and hardness of the AZ31/15 vol.% TiC composites over the AZ31 alloy is primarily responsible for their increased wear resistance at high loads. As per Archard law, harder materials show better wear resistance under similar conditions. On the other hand, a rise in normal stresses would highlight the substrate work hardening. The inclusion of TiC particles under a normal load improves the work hardening capacity of AZ31/15 vol.% TiC composites. The slope turns positive at an increased normal load, representing a shift toward more substantial wear in the main wear processes. The composite produced with FSVP surpasses the FSP variant, suggesting that superior reinforcing distribution enhances the wear characteristics. The noted decrease in specific wear rate further illustrates the efficacy of TiC reinforcement in reducing material removal under extreme working circumstances, establishing FSVP as a viable approach for wear-resistant applications.

### Wear mechanisms

**Fig. 7:**
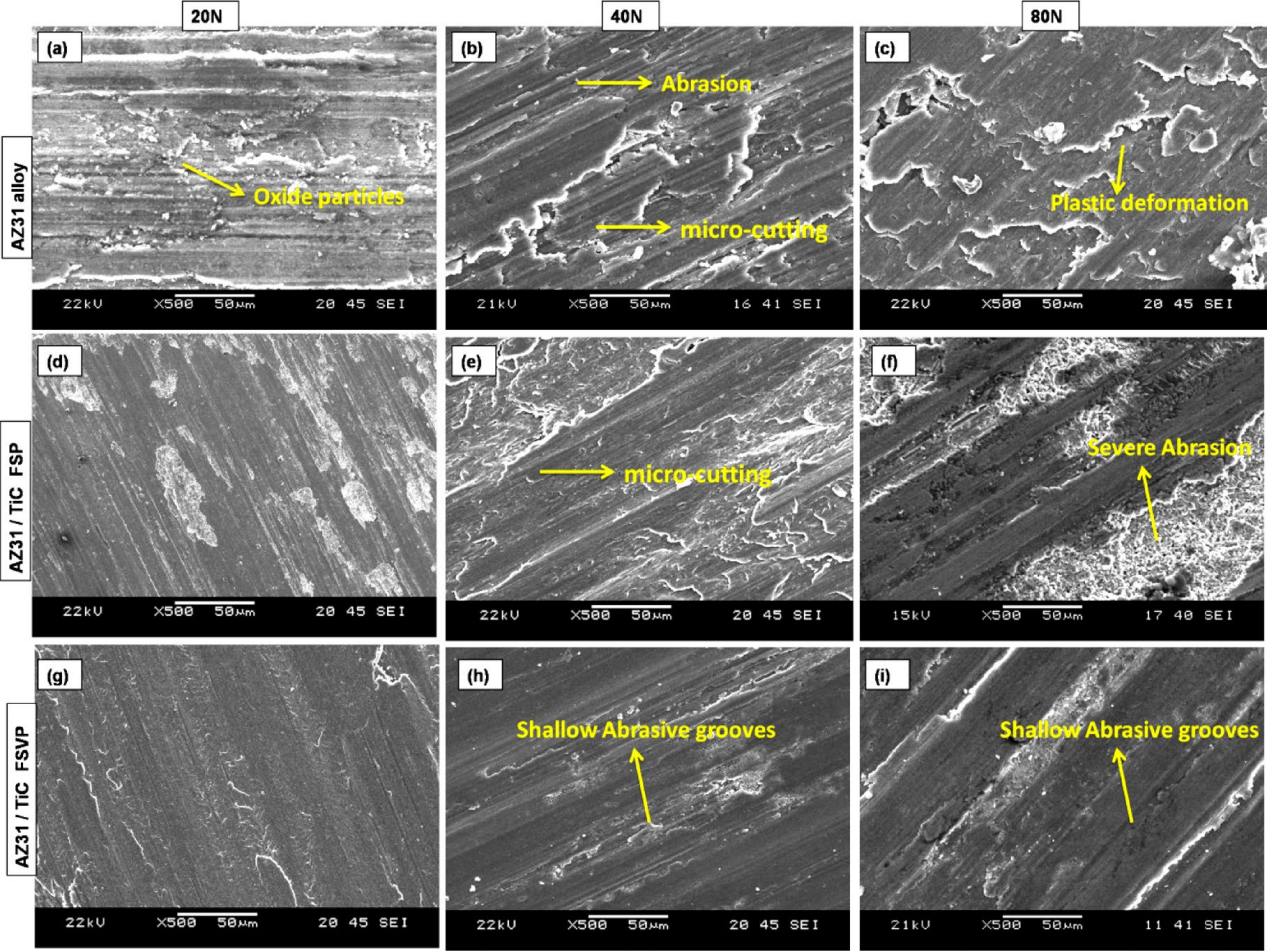
SEM micrographs of worn surfaces of (**a**–**c**) AZ31 alloy, (**d**–**f**) AZ31/TiC FSP and (**g**–**i**) AZ31/TiC FSVP subjected to various loads of 20 N to 80 N.

The eroded surfaces of the AZ31 matrix and its composites were examined by SEM images to elucidate the influence of TiC particles and the FSVP process, as well as to discern the distinct wear mechanisms present in each evaluated wear scenario. The deteriorated surface of AZ31 alloy exhibits standard oxidation and abrasion wear features, including grooves aligned with the sliding direction and scattered debris particles, at a normal load of 10–20 N (Fig. 7a). These grooves are produced by either the worn debris abrading the soft test surface (three-body abrasion) or by hard microscopic asperities on the pin surface. The magnified image in Fig. 7a shows a micro-cutting method of abrasive wear in the smallest material displacement to the sidewalls.

As seen in Fig. 7d–i, the micro-cutting in the composites is less severe and less intense than in the AZ31 alloy (Fig. 7a–c). The worn surface of the AZ31/15 vol.% TiC FSP composite has more irregular deeper and micro-cutting grooves (Fig. 7e). This can be due to fact that the weakly bonded and agglomerated TiC particles might get pulled out of the matrix and get trapped between the composite pin and the rotating disc, leading to three body wear. Whereas the FSVP processed AZ31/15 vol.% TiC composites has finer and shallower abrasive grooves. This is due to the increased hardness of FSVP composite, which provides superior resistance to cutting. In addition, the finer TiC particles act as lubricants beneath the surface of the abraded material avoiding direct contact among the surfaces due to wear and the rolling process. Consequently, FSVP composites exhibit reduced wear rates (Fig. 6) and a lower FC (Fig. 5) in contrast to the AZ31 alloy.

**Fig. 8:**
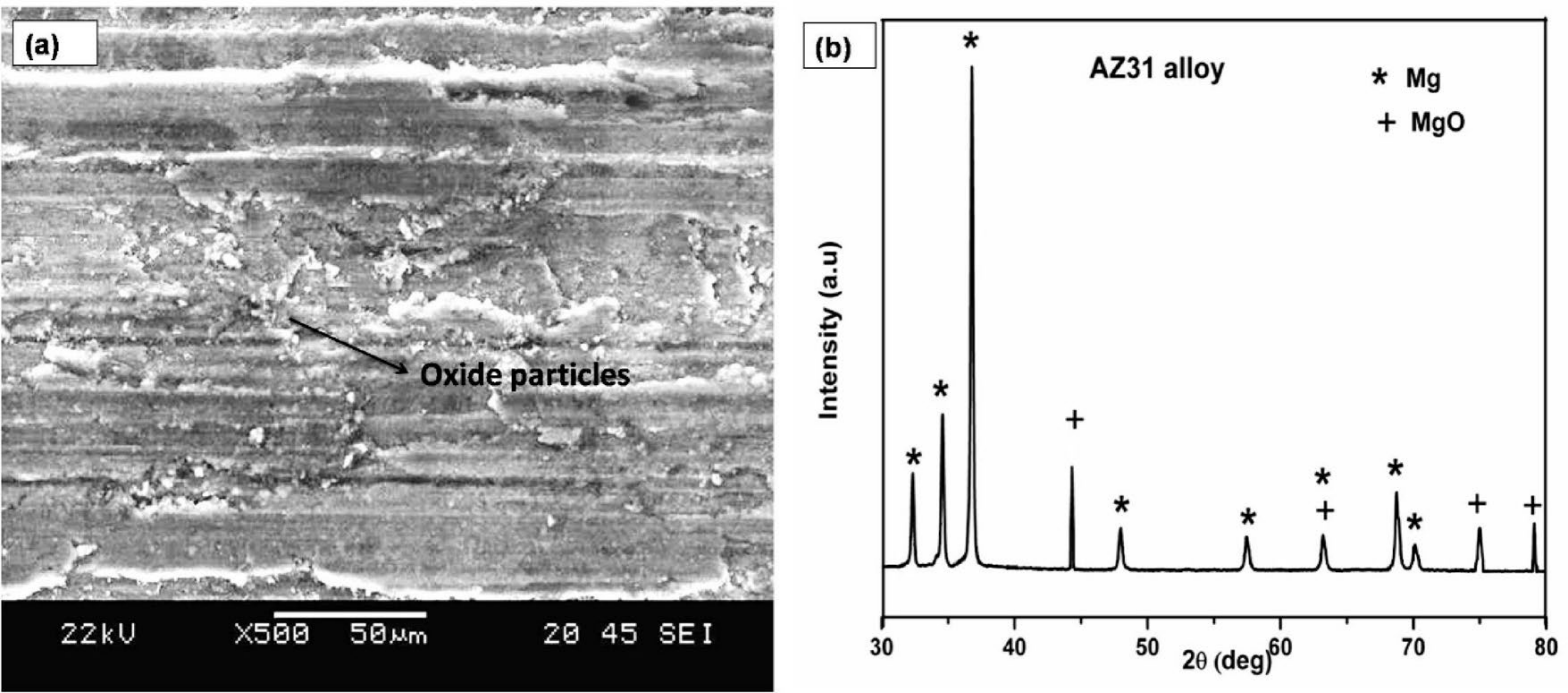
(**a**) AZ31 worn surface and (**b**) XRD of AZ31 worn surface.

When the load is increased to 20 N as shown in Fig. 6a, the wear tracks of the matrix alloy gets rougher and forms an oxide layer. The AZ31 matrix alloy experiences oxidative wear in addition to abrasive wear, which modifies the overall tribological behavior of the alloy. The worn surface of the AZ31 alloy (Fig. 7a–c) can shed additional light on the wear mechanism. The AZ31 matrix undergoes considerable distortion due to the continuous sliding action during the wear test. This subsequently results in the formation of frictional heat and the deposition of minute debris at the contact disc. This generated frictional heat causes the worn debris particles to undergo intense oxidation as Mg requires only less activation energy to get oxidized^29^ (Fig. 8a). These oxidized particles appear as tiny white dots in the alloy as seen in Fig. 7a. Thus, the formation of MgO on the surface is confirmed by XRD analysis (Fig. 8b). Moreover, because of continuous sliding motion oxide film is formed. Thus, the former tribo-induced oxide coating reduces the friction coefficient by functioning as an efficient solid lubricant^30^. Frictional heating while sliding causes oxidation of the pin surface, resulting in a reduction in the rate of wear. The oxidized debris will fill the grooves and gaps on the pin surface, creating a protective mechanical mixed layer (MML). This layer then decreases the contact between the counter disc and pin, resulting in reduced wear and friction for all the samples. In comparison to the AZ31 alloy, the AZ31-5% TiC sample (Fig. 7d–f) has a reduced fraction of oxide patches or spots and there are no similar types of oxide patches present. In composite samples, the presence of TiC particles may have resulted in reduced levels of oxidation and less frictional heating. In the composite samples, abrasion lines are seen instead of oxide spots (Fig. 7f). Abrasion causes materials to be transported without being removed from either side of the grooves^31^, thereby lowering the wear rate. The SEM images (Fig. 7g–i) of the FSVP processed AZ31-15 vol.% TiC composites shows the abraded TiC particles, indicating that these reinforcement particles abrade during the sliding motion without becoming dislodged and helping to support the imposed load and protect the matrix against deformation. This is because the TiC particles have a stronger connection with the matrix, which causes the composites to become harder and eliminate porosity. FSVP process of AZ31-15 vol.% TiC composites exhibited excellent wear resistance under all tested conditions.

## Machine learning (ML) based analysis

The ML workflow adopted in this study follows a standard supervised regression framework. The dataset comprises four input attributes: material type, applied load and sliding speed, while the specific wear rate is considered as the target variable. The material type represents a categorical variable with three levels (AZ31 alloy, AZ31–15 vol.% TiC processed by FSP and AZ31–15 vol.% TiC processed by FSVP). This categorical variable was encoded using one-hot encoding to convert it into numerical form suitable for machine learning algorithms. The dataset was randomly divided into training and testing subsets using an 80:20 split. Feature scaling was applied only to the Linear Regression model using standardization (zero mean and unit variance). Tree-based models such as Decision Tree, Random Forest and Gradient Boosting do not require feature scaling; therefore, the original feature values were used for these models.

### Linear regression

Linear regression (LR) is a statistical method used to model the relationship between a dependent variable and one or more independent variables. It does this by fitting a linear equation to the observed data. The model assumes the target variable can be expressed as a weighted linear combination of the input features, along with an error term that accounts for unexplained variability. The regression coefficients are estimated by minimizing the sum of squared differences between the predicted and observed values, usually using the ordinary least squares (OLS) method. Because of its interpretability and computational efficiency, linear regression is commonly used as a baseline model for regression problems and for studying the direct effect of individual predictors on the response variable. The linear regression model can be expressed as shown in Eq. (4).

4 $$y = \beta_{0} + \sum\limits_{i = 1}^{p} {\beta_{i} x_{i} + \varepsilon }$$

where y denotes the target variable, x_i_ represent the input features, ${\beta }_{i}$ are the regression coefficients and $\varepsilon$ is the error term.

### Decision trees

Decision Tree (DT) is a non-parametric technique of supervised learning where the relation between the variables or features and the target variable is expressed in a tree form by recursively dividing the variable space. The process of data division based on the variables forms a tree structure, consisting of nodes and leaves. The nodes define the variables and the leaves predict values, usually the average value of the target variable associated with the node. At the time of training, the decision tree identifies the split points that help minimize any predefined impurity measure, like mean squared error (MSE), thus decreasing the variances of the predicted values for each split. As the decision tree has a hierarchical representation, it easily handles non-linear and compound interactions between features.

### Random forest model

A Random Forest (RF) is an ensemble learning method that extends decision tree regression by combining the predictions of multiple independently trained decision trees to improve predictive accuracy and robustness. Every tree in the random forest regression is grown on a bootstrap sample that is drawn randomly with replacement from the total training dataset. Further, at every split in a tree, it considers a random subset of input features, thereby introducing diversity among all the trees and reducing the correlation between individual models. Each of these trees learns the nonlinear relationships between input features and target variables by recursively partitioning the feature space in a way that minimizes the prediction error. The usual criterion for this is the mean squared error of the prediction. The random forest regressor gives a final prediction by averaging the outputs of all the individual trees. This causes a reduction of variance and hence an improved generalization performance when compared to a single decision tree. It is particularly effective in the case of complex, nonlinear datasets and is robust against noise, multicollinearity and overfitting.

### Gradient boosting model

Gradient Boosting (GB) is an ensemble learning technique that builds a powerful predictive model additively by combining a set of weak learners—usually decision trees—stage-wise in a sequential manner. Unlike bagging-based ensembles, GB follows a boosting strategy where each successive model in the process is trained to correct the errors made by the previously trained models. In regression problems, GB minimizes a specified loss function, normally the MSE, by fitting new decision trees to the negative gradients, or residuals, of the loss concerning the current model predictions. At each iteration, it trains a weak regression tree on these residuals and adds to the ensemble with a learning rate controlling the contribution of each tree. The iterative optimization procedure allows the model to capture complex nonlinear relationships among input features and the target variable. The key hyperparameters of the GB Regressor are the number of estimators, learning rate and maximum tree depth-all of which work together to control model complexity and help prevent overfitting. Its ability to model complex interactions between input features and to handle heterogeneous data, GB has proved well-performing in a wide array of engineering and materials science regression tasks. The values of key hyperparameters used in the implementation of proposed study are learning_rate = 0.1, n_estimators = 200, Random state = 42 and max_depth = 4.

### Performance evaluations of various ML models

The performances of various models are evaluated based on the coefficient of determination (R^2^), calculated using Eq. (5).

5 $$R^{2} = \frac{{\sum\limits_{i = 1}^{n} {\left( {y_{i} - \hat{y}_{i} } \right)^{2} } }}{{\sum\limits_{i = 1}^{n} {\left( {y_{i} - \overline{y}_{i} } \right)^{2} } }}$$

where ŷ represents the predicted value, y denotes the actual value, ȳ signifies the mean value and n indicates the number of tests. From Eq. (5), it is evident that the value of R^2^ approaches 1 when the model completely fits the data; otherwise, it approaches 0. The R^2^ performance characteristic is significantly more effective for assessing model adequacy than other metrics such as MSE and RMSE^31^. The data obtained from the tests was divided into training and testing datasets. Out of the 135 available data points, 108 (approximately 80%) are allocated for training, while the remaining data points are set aside for validating the model. Feature standardization using the Standard Scaler method was applied only for the Linear Regression model, while tree-based models were trained using the original feature scales. The categorical variable representing material type was encoded using one-hot encoding prior to model training. The ML algorithms have been trained separately and the performance of every algorithm has been validated using statistical metrics such as RMSE, MSE, MAE and R^2^. The performance of the described algorithms in predicting the specific wear rate is shown in Table 2.

**Table 2:** Model performance summary for predicting specific wear rate.

ML Regressor	R^2^	RMSE	MSE	MAE
Linear regression	0.9444	0.0254	0.00064	0.02263
Decision tree	0.9551	0.0228	0.00052	0.02111
Random forest	0.9562	0.0225	0.00050	0.01660
Gradient boost	**0.9925**	0.0093	0.00008	0.00602

The evaluation of different ML regressors RF, DT, LR and GB shown in Table 2 provides a comprehensive understanding of their predictive performance for specific wear rate. The GB model outperforms all other models, achieving the highest R^2^ value of 0.9925, indicating an excellent fit between the actual and predicted values. It also records the lowest error values across all metrics, with an RMSE of 0.0093, MSE of 0.00008 and MAE of 0.00602. These extremely low error rates reflect the model’s superior ability to generalize and accurately predict unseen data. The performance of GB highlights the effectiveness of ensemble-based boosting techniques in capturing complex nonlinear relationships in the dataset. The RF and DT models show comparable performances, with R^2^ values of 0.9562 and 0.9551, respectively. The RMSE and MAE values for both models are closely matched, indicating that both ensemble and single-tree approaches can deliver reasonably accurate predictions. However, RF marginally outperforms the DT in all metrics, suggesting that the ensemble strategy in RF reduces variance and enhances predictive stability compared to a single tree model. LR, while still performing satisfactorily with an R^2^ value of 0.9444, exhibits the highest RMSE (0.0254), MSE (0.00064) and MAE (0.02263) among all the models. This suggests that the linear model struggles to accurately capture the underlying patterns in the data, due to the nonlinear relationships present in the system, which cannot be effectively modeled using a purely linear approach. Thus, GB emerges as the most reliable model for predicting specific wear rates, offering minimal errors and near-perfect correlation. RF and DT provide acceptable alternatives but with slightly higher prediction errors, while LR is the least effective among the methods analyzed. These results affirm the importance of choosing advanced ensemble techniques when dealing with complex material behavior in tribological studies.

**Fig. 9:**
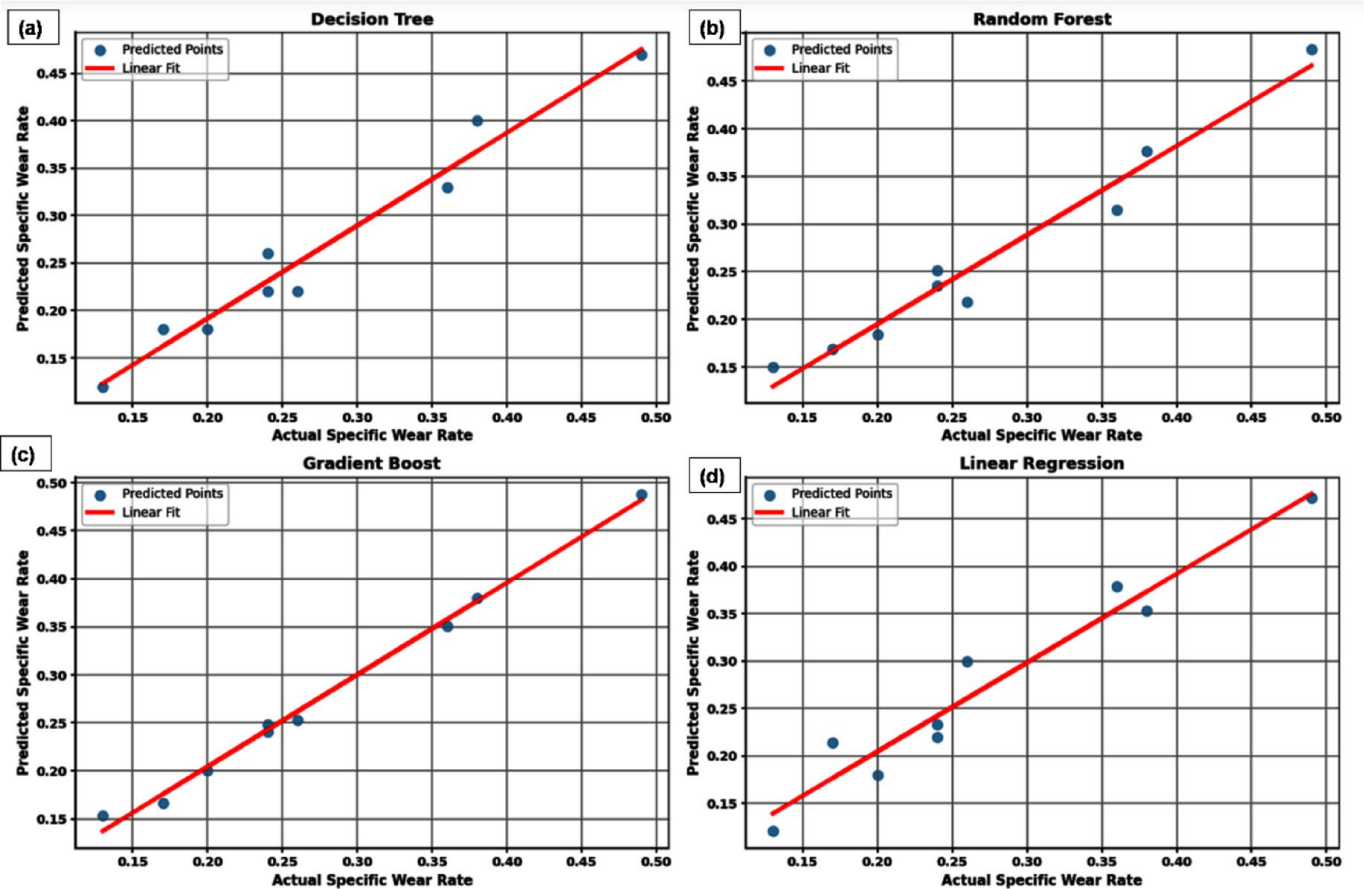
Actual vs. predicted specific wear rate plots for various ML models (**a**) DT, (**b**) RF, (**c**) GB and (**d**) LR.

Figure 9 shows the actual vs. predicted specific wear rate plots for the ML models (a) DT, (b) RF, (c) GB and (d) LR, respectively. It provides significant insights into the predictive capability of each model. In all cases, a positive correlation between the actual and predicted values is observed and is indicated by the clustering of data points along the red linear fit line. The GB (Fig. 9c) model demonstrates the best predictive performance among the models considered. The projected points closely correspond to the actual values, exhibiting low divergence from the linear trend line. This suggests that GB effectively captures the underlying nonlinear relationships within the dataset, leading to higher prediction accuracy.

The RF (Fig. 9b) model also shows strong predictive performance, comparable to that of GB. The distribution of points around the linear fit is tight and the model effectively generalizes the data without significant overfitting or underfitting. This can be attributed to RF ensemble approach, where multiple decision trees work collectively to enhance robustness and reduce variance. The DT (Fig. 9a) model, although showing a reasonable correlation, displays slightly higher scattering compared to GB and RF. This is expected, as a single DT is more prone to overfitting and less capable of handling complex data patterns compared to ensemble methods.

On the other hand, the LR (Fig. 9d) model exhibits the largest deviation of predicted values from the actual measurements. The scattering of points is more pronounced, indicating that a simple linear relationship is insufficient to capture the complexity of the wear rate behavior. LR assumes a straight-line relationship, which may not be adequate for the given data where interactions and nonlinearities are present. Comparatively, ensemble learning methods such as RF and GB outperform both the single DT and LR models. This highlights the importance of using advanced ML algorithms, particularly ensemble-based approaches, when modeling and predicting complex material behavior such as specific wear rates.

### Feature importance analysis for the various ML techniques

**Fig. 10:**
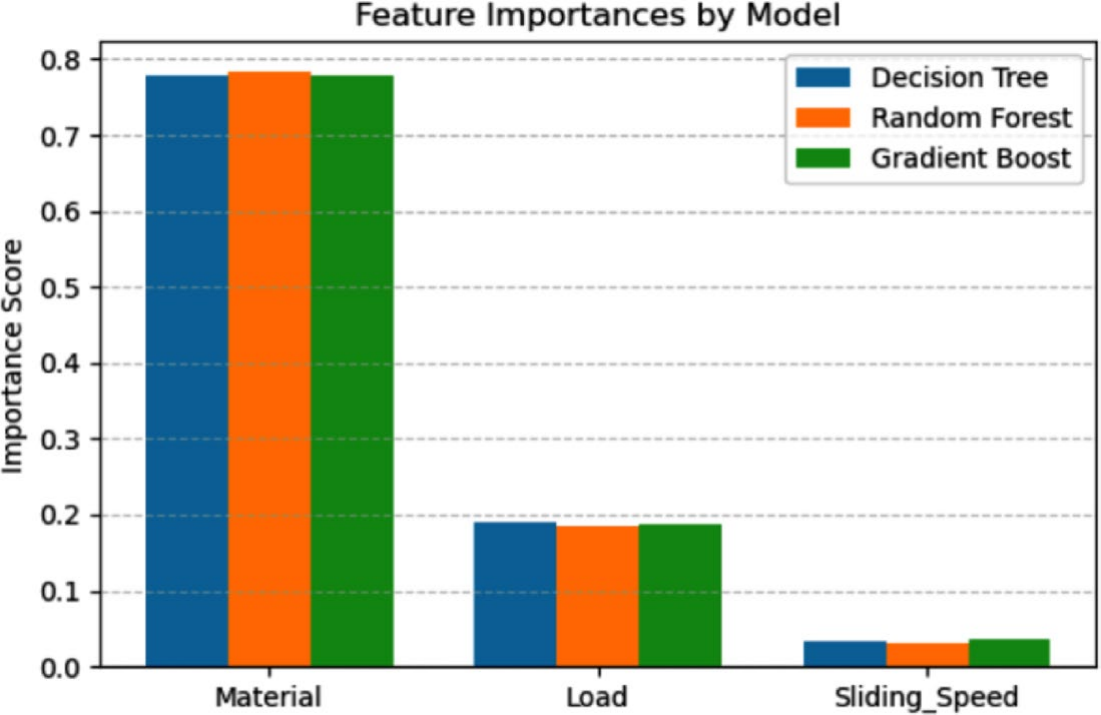
Feature importance analysis for the various ML techniques.

Figure 10 shows the feature importance analysis for the DT, RF and GB models provides critical insights into the influence of different input parameters on the prediction of specific wear rate. Across all three models, the material parameter exhibits the highest importance score, contributing approximately 78% to the predictive capability. This indicates that the material type governs the specific wear rate behavior, emphasizing its critical role in controlling tribological performance. The consistent dominance of the material feature across DT, RF and GB models further validates its fundamental impact, independent of the modeling technique employed. The load parameter holds the second-highest importance, contributing around 18–19% to the model outputs. Although significantly lower than the material contribution, the load still plays a substantial role in influencing the wear behavior. This aligns with classical tribological theory, where increasing normal load typically accelerates material removal rates.

The sliding speed parameter shows the least importance, contributing less than 5% across all models. This suggests that, within the experimental conditions studied, variations in sliding speed exert a minimal effect on specific wear rate compared to material and load. The consistently low importance of sliding speed across all models implies that it may be treated as a secondary factor when optimizing the wear performance.

### Residual plots for ML models

**Fig. 11:**
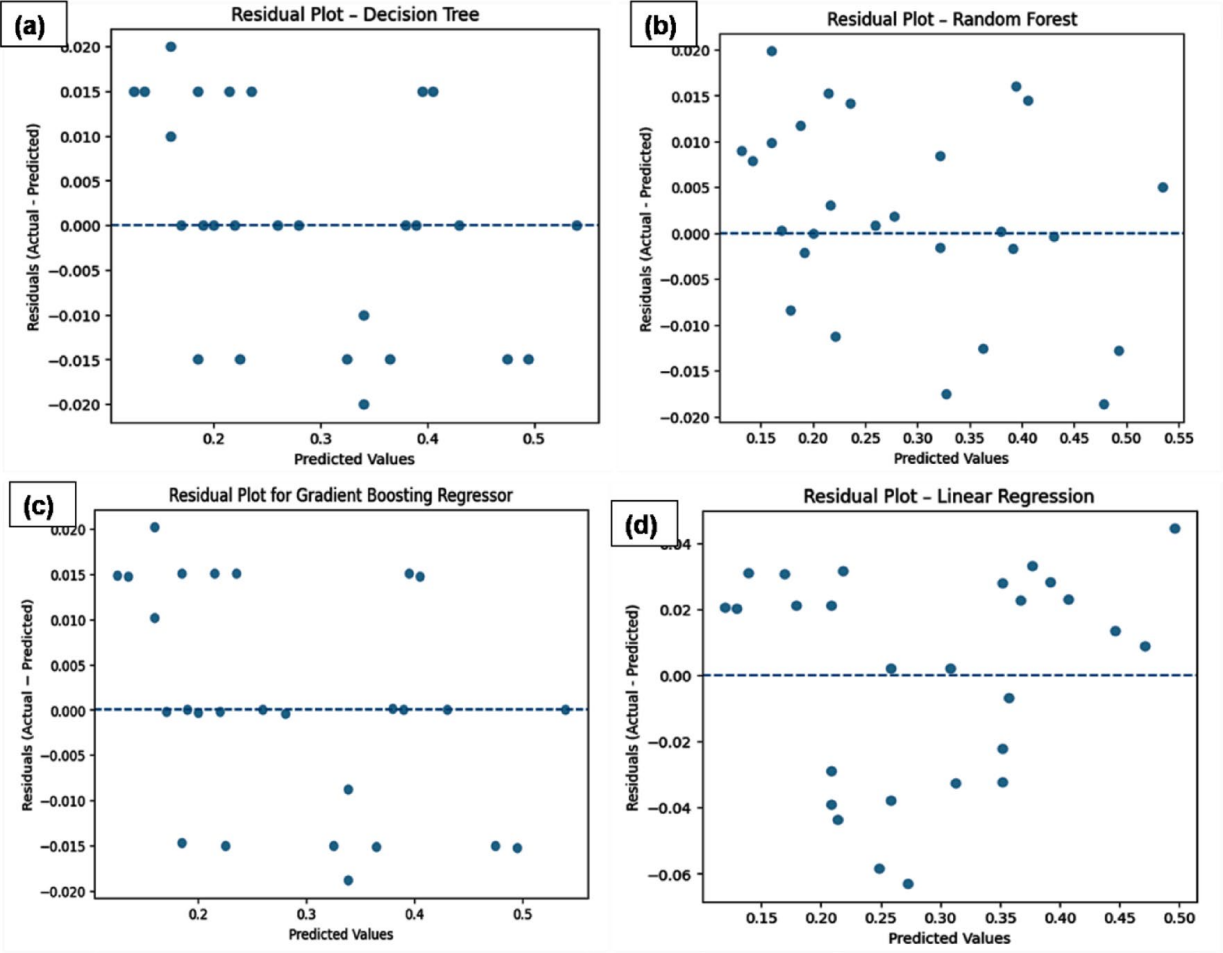
Residual plots of ML models (**a**) DT, (**b**) RF, (**c**) GB and (**d**) LR.

The residual plots of ML models are shown in Fig. 11: (a) DT, (b) RF, (c) GB and (d) LR, respectively. Residual plots are used to evaluate the adequacy and reliability of regression models by examining the distribution of prediction errors.

As shown in Fig. 11b, the RF residuals are evenly scattered around zero over the full range of predicted values. This means that the model does a decent job capturing the underlying non-linear relationships, but some spread of residuals at higher predicted values shows some uncertainty about the predictions. The DT (Fig. 11a) has a unique lump of residuals at some predicted values, a feature of tree-based single estimators. A sequence of zero residuals and quite large positive and negative spikes suggest that the model is overfitting a few training samples and cannot generalize well, particularly with unseen data. Conversely, the LR (Fig. 11d) model has an obvious pattern in the residuals, with greater spread and a consistent drift away from zero. This non-random structure shows that a purely linear assumption breaks down for this data. It also shows that the model fails to capture the non-linear interactions between inputs. The GB (Fig. 11c) regressor shows the best residual behavior because the residuals are very tight around zero and there is slight variation between predicted values. Its lack of clear trend or heteroscedasticity means the bias–variance trade-off is well balanced and the model generalizes well. The boosting strategy can predict well and still recover intricate interactions.

### Training and validation curves

**Fig. 12:**
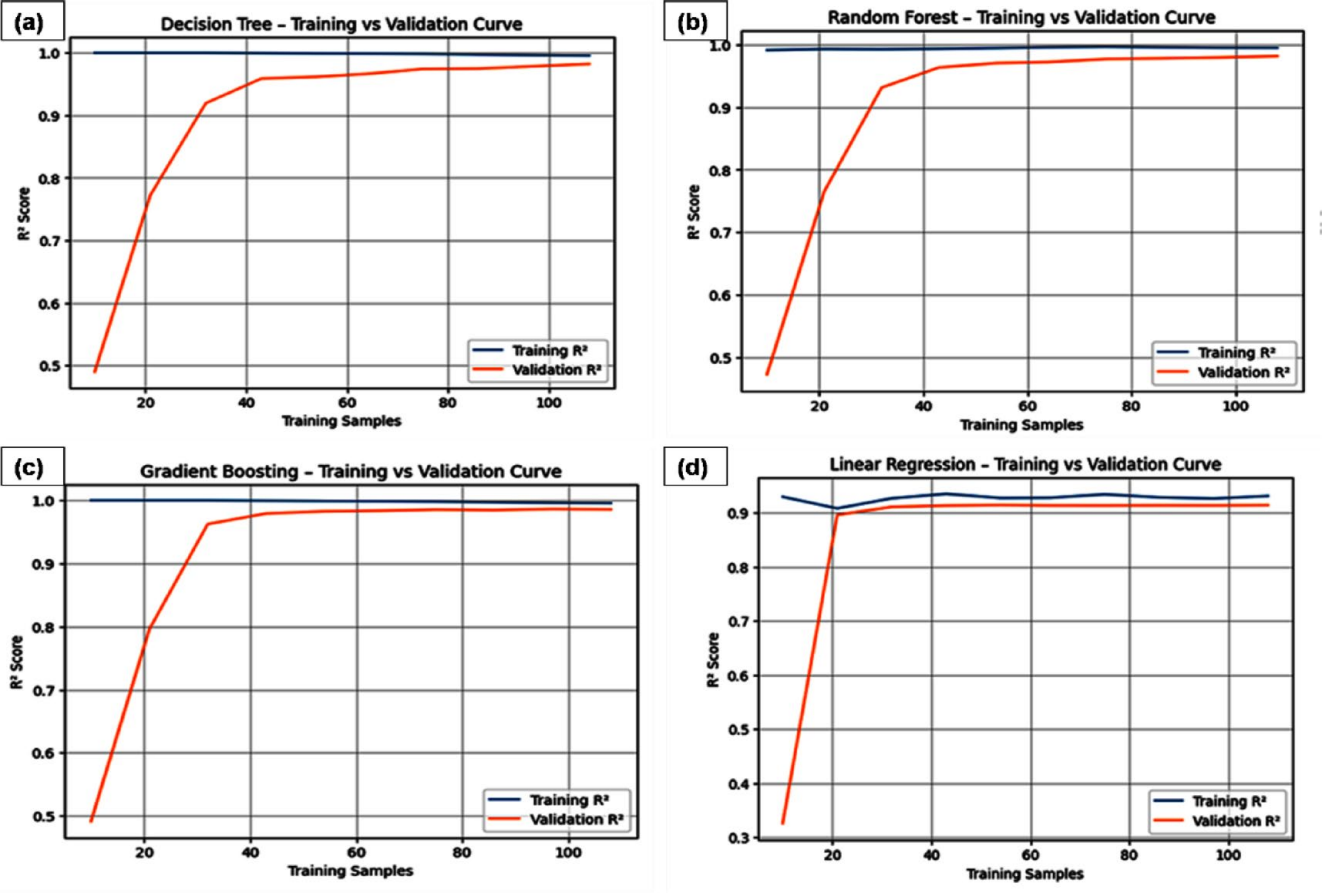
Learning and validation curves of ML models (**a**) DT, (**b**) RF, (**c**) LR and (**d**) GB.

The training and validation curves of ML Models used in this study are shown on Fig. 12. These curves are widely used as means of checking a model’s generalization capability and diagnosing whether it is underfitting or overfitting. These curves convey related information about model complexity, data adequacy and bias-variance tradeoff by showing the way in which training and validation errors evolve with either size of training data or specific values of hyperparameters. The training-validation learning curves of ML models are shown in Fig. 12: (a) DT, (b) RF, (c) LR and (d) GB, respectively. It can be noted that all models possess stable and well-generalized predictive behavior for growing training data sizes. LR (Fig. 12c) shows closely overlapping training and validation R^2^ values, ensuring strong generalization capabilities but poor nonlinear modeling capacity. For the DT model (Fig. 12a), there are initially high variances for smaller training data sizes; yet, as both training and validation performances converge with an increasing number of data, it is confirmed that the model has suppressed overfitting. Amongst all models, the ensemble models of RF (Fig. 12b) and GB (Fig. 12d) possess the most robust output performances, with negligibly differences between the training and validation curves throughout the data range, signifying strong variance control and generalization capabilities. GB achieves consistently high validation R^2^ values, confirming its superior ability to model complex nonlinear interactions without overfitting and validating that the reported high predictive performance arises from genuine data-driven learning rather than model memorization.

### Input parameter correlation matrix

**Fig. 13:**
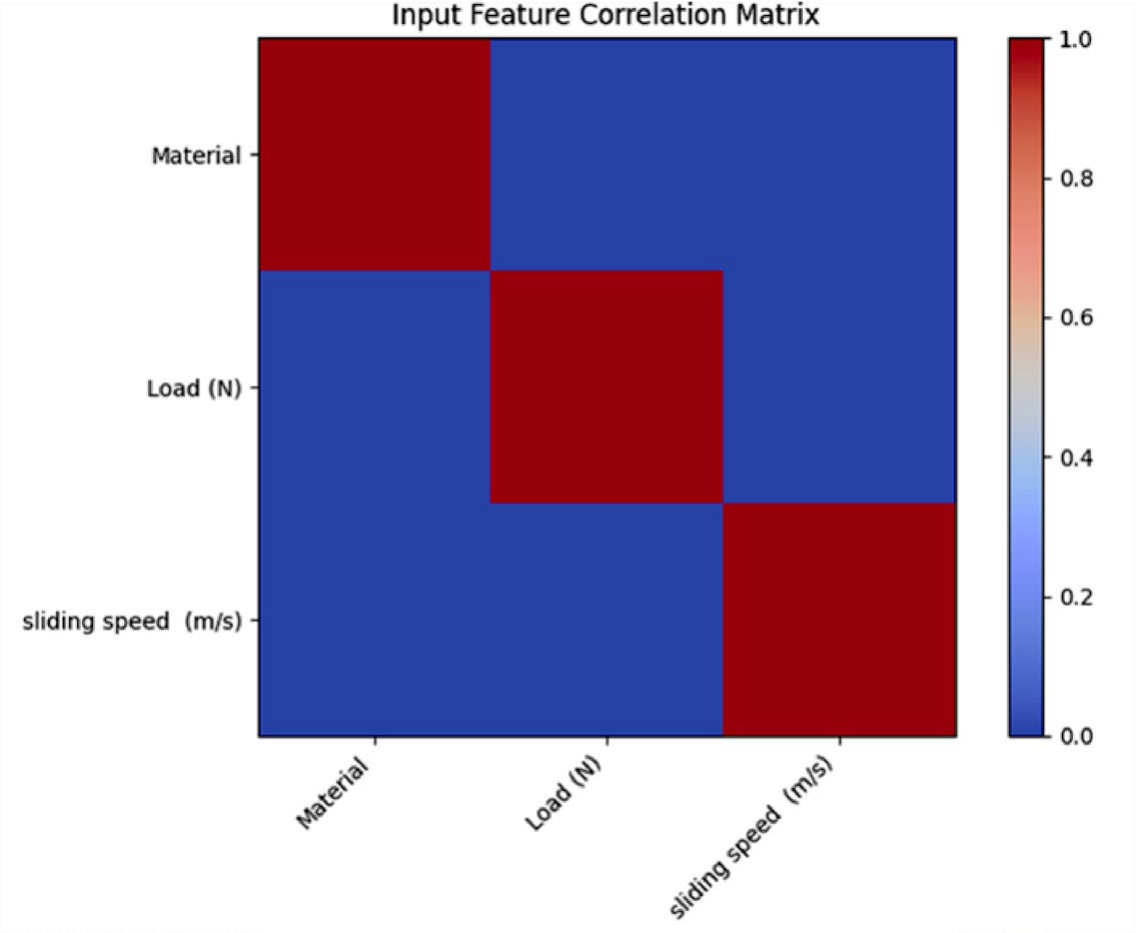
Correlation matrix of input parameters.

Figure 13 presents the Pearson correlation matrix of the input features, namely material, applied load (N) and sliding speed (m/s).

In Fig. 13, the diagonal elements have unit correlation, but the off-diagonal elements do show a negligible linear correlation between the input parameters. More importantly, the correlation coefficients between material versus load, material versus sliding speed and load versus sliding speed are close to zero, confirming that there is no strong linear dependence between the features. Therefore, these low levels of inter-feature correlation further imply that most of the chosen input variables are independent, thus minimizing multicollinearity issues in the subsequent machine learning models. As a result, each of the input parameters contributes distinct information to the prediction task, supporting the robustness and interpretability of the developed models.

### SHAP analysis

SHAP (SHapley Additive exPlanations) analysis was employed to enhance the interpretability of the developed ML models by quantifying the contribution of each input feature to the model predictions. Unlike the traditional feature-importance measures, SHAP presents both global and local explanations based on cooperative game theory, thus having consistent and additive attributions.

**Fig. 14:**
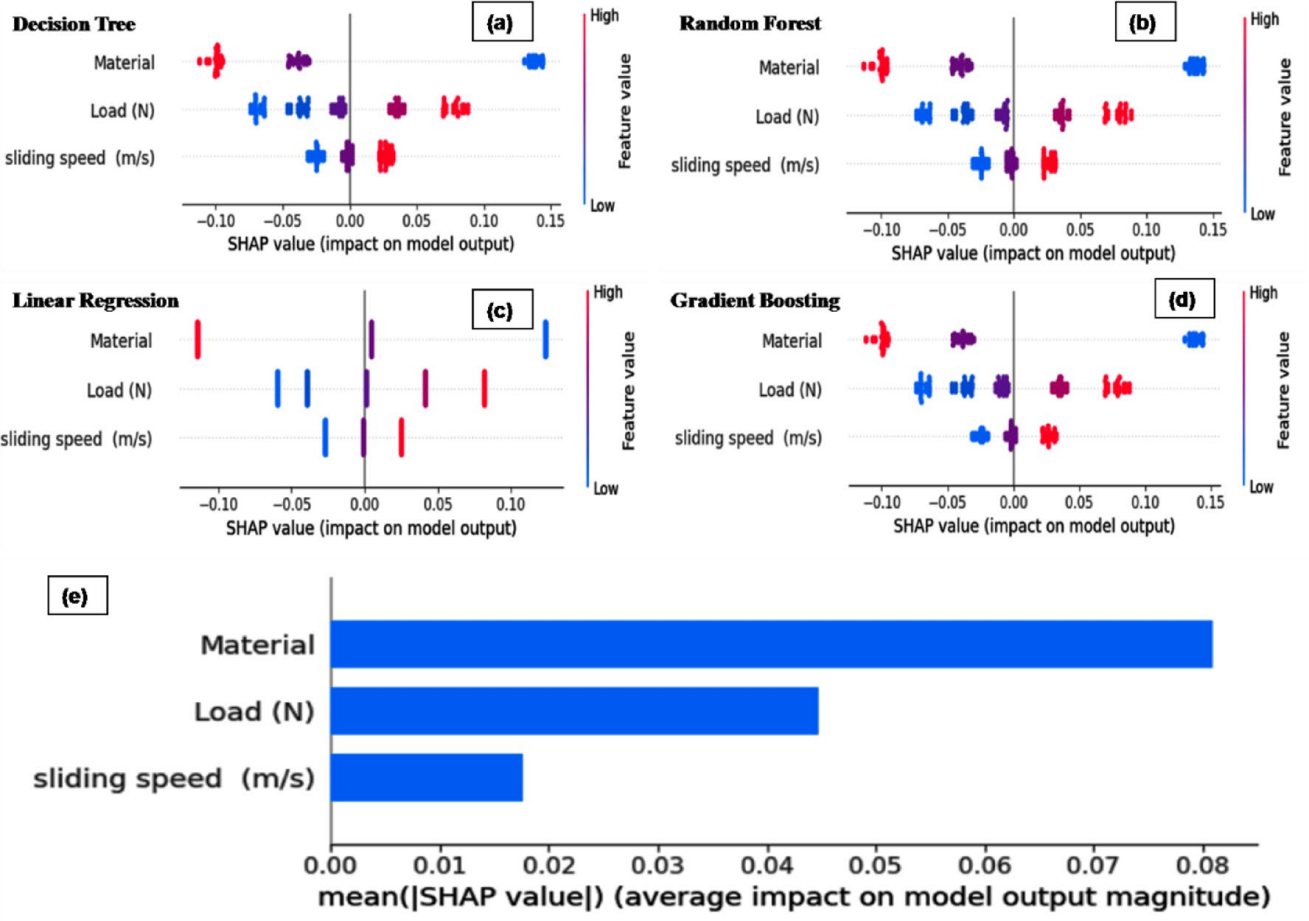
SHAP analysis of ML models (**a**) DT, (**b**) RF, (**c**) LR, (**d**) GB and (**e**) contribution of each variable to the prediction wear rate prediction.

Figure 14. shows the SHAP analysis of ML models (a) DT, (b) RF, (c) LR, (d) GB and (e) contribution of each variable to the prediction wear rate prediction. The beeswarm plots of SHAP summaries show that material is the most important predictor in all models, with the highest SHAP value range and a systematic positive/negative contribution based on the magnitude, thus validating its significant contribution to the response of the model. The second most important predictor is the applied load, with higher values leading to a systematic increase in the predicted response and sliding speed has lower magnitudes of SHAP, indicating a less significant, yet non-negligible, contribution, due to interaction and nonlinear effects modelled by tree-based and boosting models. The model-independent and systematic ordering of predictors shown by the results of linear and ensemble models validate the strong, non-spurious and interpretable contributions of feature predictors to systematic model predictions, while the broader range of SHAP values shown by the results of the RF (Fig. 14b) and GB (Fig. 14d) models validate the superior capabilities of the models in capturing complex, non-spurious and systematic relationships between predictors and model predictions.

SHAP values are symmetrical and bound in a tight range for all features in LR. Although material and load still trump sliding speed, the small SHAP values show limited explanatory power. This behavior reflects the linear model’s incapacity to model complex and non-linear interactions among the process parameters. The Gradient Boosting model has the most structured and physically meaningful SHAP patterns. Material is the leading parameter with consistently elevated SHAP values, then load, with sliding speed providing a secondary, yet noticeable contribution. The clean partitioning of high and low feature values across positive and negative SHAP regions confirms the model learns non-linear interactions and hierarchical feature dependencies. The global SHAP importance ranking reinforces these findings, ranking material as the highest parameter controlling model output, then applied load and sliding speed. This ranking also accords with empirical tribological wisdom, in which material plays the leading role in wear characteristics and load and sliding velocity are secondary.

Figure 14e also shows the importance of variables in the regression model based on Gradient Boosting, analyzed through the mean absolute SHAP values. From the bar graph, the contribution of each variable to the prediction of the wear rate is indicated. Of all the variables considered, material is the one that has the highest importance in determining the model output, followed by load and the least is sliding speed. An important conclusion that can be drawn is that material composition is the most dominant factor that determines the component’s performance, whereas the other factors are also considered.

### Limitations of this study

Despite the promising results obtained in this work, several limitations and sources of uncertainty must be considered. First, experimental uncertainties arise from inherent material heterogeneity, variations in reinforcement particle distribution and limitations in measurement repeatability, all of which may introduce variability in the observed tribological responses. Second, process-related uncertainties are associated with localized thermal and mechanical fluctuations during processing, particularly under vibration-assisted conditions, which can influence microstructural evolution and, consequently, wear behavior. On the third aspect, some modeling uncertainties are considered due to the limited size of the set of data, correlations among the features, or the simplifications included in the ML models used. These factors may affect the generalizability of the developed models beyond the investigated parameter space. Addressing these limitations through larger, more diverse datasets, extended process parameter ranges and the integration of physics-informed or uncertainty-aware learning frameworks constitutes an important direction for future research.

## Conclusions

The impact of TiC addition on the magnesium alloy AZ31 tribological performance under varied applied loads was investigated. TiC particles were found to be effectively dispersed and bonded to the matrix using the FSVP process. The FSVP-processed AZ31/15 vol.% TiC composites exhibited extremely low porosity levels. Compared to the matrix alloy and FSP samples grain size, samples processed with FSVP showed significant grain refinement. FSVP AZ31/15 vol.% TiC composites exhibited the highest hardness of about 114 HV with 83% improvement compared to AZ31 alloy. The use of TiC particles reduced the friction coefficient and enhanced the wear resistance of composites, especially at elevated applied loads. The adhesion and plastic deformation has been mitigated to a larger extent by encapsulation of TiC using FSVP and mechanisms was found to change (transitioned) to abrasion from adhesion/delaminations/plastic deformations. The GB model outperforms all other regressors with the highest R^2^ value (0.9925) and the lowest error metrics (RMSE, MSE, MAE), establishing its superiority in predictive accuracy. The feature importance analysis reveals that the material type overwhelmingly governs the wear rate, contributing approximately 78% across all models. Load exerts a secondary yet considerable influence (18–19%), while sliding speed plays a minimal role (> 5%). The ML analysis performed in this study demonstrates that data-driven regression models can reliably capture the complex, non-linear relationships governing specific wear rate in the investigated material system. Specifically, among the evaluated regression models, GB consistently exhibited superior predictive performance, clearly pointing to its effectiveness in approximating complex dependencies between material, applied load and sliding speed for a given material pair. Finally, material and applied load were found to significantly affect a particular rate of wear, while sliding speed has a small impact, a fact that further underlines their effectiveness in this field. Although the results are encouraging, experimental variability, process-induced fluctuations and data- and model-related uncertainties limit the generalizability of the findings, highlighting the need for larger datasets and more robust, physics-informed modeling in future studies.

## Data Availability

The datasets generated and/or analyzed during the current study are available from the corresponding author upon request.

## References

[1] Miracle, D. B. Metal matrix composites from science to technological significance. *Compos. Sci. Technol.* **65**, 2526–2540. 10.1016/j.compscitech.2005.05.027 (2005).

[2] Abbas, A., Rajagopal, V. & Huang, S.-J. Magnesium metal matrix composites and their applications. *Magnes. Alloy Struct. Prop. Intech Open*10.5772/intechopen.96241 (2022).

[3] Ye, H. Z. & Liu, X. Y. Review of recent studies in magnesium matrix composites. *J. Mater. Sci.* **39**, 6153–6171. 10.1023/B:JMSC.0000043583.47148.31 (2004).

[4] Luo, A. A. Magnesium casting technology for structural applications. *J. Magnes. Alloys* **1**, 2–22. 10.1016/j.jma.2013.02.002 (2013).

[5] Saranu, R., Chanamala, R. & Putti, S. Review of magnesium metal matrix composites. *IOP Conf. Ser. Mater. Sci. Eng.* **961**, 12001. 10.1088/1757-899X/961/1/012001 (2020).

[6] Katiyar, J. K., Al Hammad, J. & Mohammed, A. S. *Tribological Properties of Light Metal Matrix Composites* (Elsevier Ltd, 2021). 10.1016/B978-0-12-819724-0.00104-X.

[7] Zhang, M. et al. Achieving high mechanical and wear properties in the AZ31/(CeO2+ZrO2)p surface composite using friction stir processing: Application of vibration. *Vacuum* **218**, 112654. 10.1016/j.vacuum.2023.112654 (2023).

[8] Bagheri, B., Abdollahzadeh, A., Sharifi, F. & Abbasi, M. The role of vibration and pass number on microstructure and mechanical properties of AZ91/SiC composite layer during friction stir processing. *Proc. Inst. Mech. Eng. C* **236**, 2312–2326. 10.1177/09544062211024281 (2022).

[9] Zhou, Z. et al. Effect of vibration on mechanical and tribological characteristics during friction stir processed FeAlCrMoNb high entropy alloys particle reinforced AZ 31 Mg alloy. *Arch. Civ. Mech. Eng.* **24**, 183. 10.1007/s43452-024-00995-6 (2024).

[10] Bagheri, B. & Abbasi, M. Development of AZ91/SiC surface composite by FSP: Effect of vibration and process parameters on microstructure and mechanical characteristics. *Adv. Manuf.* **8**(1), 82–96. 10.1007/s40436-019-00288-9 (2020).

[11] Singla, S., Kang, A. S. & Sidhu, T. S. Development and characterization of WE43/nano-TiC surface composite by friction stir processing technique. *Meas. Control* **53**, 730–741. 10.1177/0020294019895302 (2020).

[12] M, K. B. et al. Coated and uncoated reinforcements metal matrix composites characteristics and applications-a critical review. *Cogent Eng.*10.1080/23311916.2020.1856758 (2020).

[13] Contreras, A., Lopez, V. H. & Bedolla, E. Mg/TiC composites manufactured by pressureless melt infiltration. *Scr. Mater.* **51**, 249–253. 10.1016/j.scriptamat.2004.04.007 (2004).

[14] Kaliyaperumal, G. et al. Experimental study and TiC interfacial action on microstructural and mechanical properties of AZ31 alloy composite made by stir casting route. *Mater. Today Proc.*10.1016/j.matpr.2023.06.131 (2023).

[15] Aydin, F., Sun, Y. & Turan, M. E. E. Influence of TiC content on mechanical, wear and corrosion properties of hot-pressed AZ91/TiC composites. *J. Compos. Mater.* **54**, 141–152. 10.1177/0021998319860570 (2020).

[16] Kumar, A., Kumar, S., Mukhopadhyay, N. K., Yadav, A. & Sinha, D. K. Effect of TiC reinforcement on mechanical and wear properties of AZ91 matrix composites. *Int. J. Metalcast.* **16**, 2128–2143. 10.1007/s40962-021-00747-9 (2022).

[17] Mukunda, S. G., Srivastava, A., Boppana, S. B., Dayanand, S. & Yeshwanth, D. Wear performance prediction of MWCNT reinforced AZ31 composite using machine learning technique. *J. Bio Tribo Corros.* **9**, 27. 10.1007/s40735-023-00745-w (2023).

[18] Ammisetti, D. K. & Kruthiventi, S. S. H. Experimental investigation of the influence of various wear parameters on the tribological characteristics of AZ91 hybrid composites and their machine learning modeling. *J. Tribol.* **146**(5), 051704. 10.1115/1.4064397 (2024).

[19] Sheikh, K. A., Khan, M. M., Roga, S. & Qureshi, T. Modeling of wear behavior in Al5052/cenosphere composites using machine learning. *J. Tribol.* **148**(1), 011403. 10.1115/1.4068679 (2026).

[20] Satish Kumar, T., Thankachan, T., Čep, R. & Kalita, K. Characterisation of AZ31/TiC composites fabricated via ultrasonic vibration assisted friction stir processing. *Sci. Rep.* **14**, 26686. 10.1038/s41598-024-77814-8 (2024). 39496743 10.1038/s41598-024-77814-8 PMC11535567

[21] Thavamani, R., Balusamy, V., Nampoothiri, J., Subramanian, R. & Ravi, K. R. Mitigation of hot cracking in Inconel 718 superalloy by ultrasonic vibration during gas tungsten arc welding. *J. Alloys Compd.* **740**, 870–878. 10.1016/j.jallcom.2017.12.295 (2018).

[22] Manroo, S., Khan, N. & Ahmad, B. Study on surface modification and fabrication of surface composites of magnesium alloys by friction stir processing: A review. *J. Eng. Appl. Sci.* **69**, 25. 10.1186/s44147-022-00073-9 (2022).

[23] Kumar, T. S., Shalini, S. & Thankachan, T. Friction stir processing-based surface modification of AZ31 magnesium alloy. *Mater. Manuf. Process,* **38**, 1426–1435. 10.1080/10426914.2023.2165670 (2023).

[24] Patil, N. A., Pedapati, S. R., Mamat, O. & Lubis, A. M. H. S. Morphological characterization, statistical modeling and wear behavior of AA7075-titanium carbide-graphite surface composites via friction stir processing. *J. Mater. Res. Technol.* **11**, 2160–2180. 10.1016/j.jmrt.2021.02.054 (2021).

[25] Moharrami, A. et al. Enhancing the mechanical and tribological properties of Mg2Si-rich aluminum alloys by multi-pass friction stir processing. *Mater. Chem. Phys.* **250**, 123066. 10.1016/j.matchemphys.2020.123066 (2020).

[26] Jolokhani, A., Razaghian, A., Moharami, A. & Emamy, M. Microstructure and tribological properties of as-cast and multi-pass friction stir processed Mg-0.5Zn-0.5Zr/SiC composite fabricated by stir casting technique. *J. Mater. Res. Technol.* **27**, 7823–7838. 10.1016/j.jmrt.2023.11.205 (2023).

[27] Singh, A. & Bala, N. Fabrication and tribological behavior of stir cast Mg/B4C metal matrix composites. *Metall. Mater. Trans. A* **48**, 5031–5045. 10.1007/s11661-017-4203-x (2017).

[28] Sankar, C., Gangatharan, K., Singh, S. C. C. E. & Sivaraj, M. Influence of AZ91 alloy reinforced with nano B4C particles on microstructural characterization, hardness and tribological properties prepared through powder metallurgy. *Mater. Res. Express*10.1088/2053-1591/ac2ce5 (2021).

[29] Krishna, S. A., Radhika, N., Saleh, B. & Manivannan, S. Microstructural mechanical and corrosion properties of SS304/HEA surface layer produced by friction stir processing. *J. Alloys Compd.* **953**, 170153. 10.1016/j.jallcom.2023.170153 (2023).

[30] Moharami, A. & Qodosi, P. Enhanced dry sliding friction and wear behaviors of Mg–Mg_2_Si composites. *Compos. Commun.* **36**, 101365. 10.1016/j.coco.2022.101365 (2022).

[31] Ragunath, S., Radhika, N., Krishna, S. A. & Rajeshkumar, L. A study on microstructural, mechanical properties and optimization of wear behavior of friction stir processed AlCrCoFeNi high entropy alloy reinforced SS410 using response surface methodology. *Heliyon* **10**, e24429. 10.1016/j.heliyon.2024.e24429 (2024). 38293432 10.1016/j.heliyon.2024.e24429 PMC10826323

